# Correction: Moon, D.O. Curcumin as a Dual Modulator of Pyroptosis: Mechanistic Insights and Therapeutic Potential. *Int. J. Mol. Sci.* 2025, *26*, 7590

**DOI:** 10.3390/ijms27031464

**Published:** 2026-02-02

**Authors:** Dong Oh Moon

**Affiliations:** Department of Biology Education, Daegu University, 201, Daegudae-ro, Gyeongsan-si 38453, Gyeongsangbuk-do, Republic of Korea; domoon@daegu.ac.kr; Tel.: +82-53-852-6992

After the publication of the review article [1], one of the referenced manuscripts was found to have been retracted. To enhance the quality and scientific rigor of the manuscript, the author decided to remove the following reference number 95 from the reference list.

95. Li, Y.; Sun, W.; Han, N.; Zou, Y.; Yin, D. Curcumin inhibits proliferation, migration, invasion and promotes apoptosis of retinoblastoma cell lines through modulation of miR-99a and JAK/STAT pathway. *BMC Cancer* **2018**, *18*, 1230.

In line with this revision, all text describing the involvement of the JAK/STAT signaling pathway that was solely supported by this reference has also been deleted to ensure the accuracy and integrity of the manuscript. As a result, the numbering of the remaining references has been rearranged accordingly. The authors state that the scientific conclusions are unaffected. This correction was approved by the Academic Editor. The original publication has also been updated.
